# NK Cells Activated through Antibody-Dependent Cell Cytotoxicity and Armed with Degranulation/IFN-γ Production Suppress Antibody-dependent Enhancement of Dengue Viral Infection

**DOI:** 10.1038/s41598-018-36972-2

**Published:** 2019-02-01

**Authors:** Peifang Sun, Maya Williams, Nishith Nagabhushana, Vihasi Jani, Gabriel Defang, Brian J. Morrison

**Affiliations:** 1 Henry Jackson Foundation, Bethesda, MD USA; 2 Viral and Rickettsial Diseases Department, Infectious Diseases Directorate, Naval Medical Research Center, Silver Spring, MD USA

## Abstract

Antibody (Ab)-dependent enhancement (ADE) is a hypothesized mechanism of increased disease severity during secondary dengue virus (DENV) infection. This study investigates Ab-dependent cell cytotoxicity (ADCC) in counteracting ADE. In our system, DENV and DENV-immune sera were added to peripheral blood mononuclear cells (PBMCs), and ADE and NK cell activation were simultaneously monitored. ADE was detected in monocytes and a concurrent activation of NK cells was observed. Activated NK cells expressed IFN-γ and CD107a. IFN-γ was detected at 24 hours (24 h) followed by a rapid decline; CD107a expression peaked at 48 h and persisted for >7 days. Optimal activation of NK cells required the presence of enhancement serum together with ADE-affected monocytes and soluble factors, suggesting the coexistence of the counteractive ADCC Abs, in the same ADE-serum, capable of strongly promoting NK cell activation. The function of NK cells against ADE was demonstrated using a depletion assay. NK cell-depleted PBMCs had increased ADE as compared to whole PBMCs. Conversely, adding activated NK cells back into the NK-depleted-PBMCs or to purified monocytes decreased ADE. Blocking IFN-γ expression also increased ADE. The study suggests that under ADE conditions, NK cells can be activated by ADCC Abs and can control the magnitude of ADE.

## Introduction

Dengue virus (DENV), a single stranded RNA virus in the genus *flavivirus*, is comprised of four closely related but antigenically distinct serotypes, DENV-1, -2, -3, and -4. All four serotypes can cause disease in humans, with severity ranging from mild dengue fever (DF) to severe dengue haemorrhagic fever (DHF), and to the life-threatening dengue shock syndrome (DSS)^1,2^. DHF/DSS, severe dengue disease, represents 2.5 to 20% of total hospitalized dengue cases, and is responsible for 20,000 deaths every year^3^.

Primary DENV infection with one serotype usually results in non-complicated DF and the development of both humoral and cellular immunity, which protects against reinfection from the same serotype for a life-long term. However, this immune response does not confer long-term cross-protection to other serotypes. Instead, previous exposure to DENV is associated with risks of severe DENV disease^4–6^ during a heterologous secondary infection through a hypothesized mechanism known as antibody (Ab)-dependent enhancement (ADE). ADE suggests that when DENV escapes from neutralizing Ab, it establishes a higher level of infection in Fc receptor (FcR)-bearing cells through FcR-facilitated binding and internalization of Ab-virus complex. ADE can lead to a higher number of infected cells (extrinsic ADE), as well as altered immune response in infected cells (intrinsic ADE) resulting in increased virus production on the per cell basis^7^. ADE has been also demonstrated *in vivo* in mice^8^ and in nonhuman primates^9^ resulting in increased clinical manifestation and viremia. Therefore, non-neutralizing Abs pose a great concern for vaccine development and seeking a mechanism to combat against ADE is an urgent priority.

Our group recently reported that non-neutralizing sera from a group of endemic subjects previously infected with DENV can bind to the surface of infected cells and can lead to rapid NK cell degranulation^10^, demonstrating the existence of Abs, in the same sera, capable of triggering Ab-dependent cell cytotoxicity (ADCC). The critical role of ADCC in controlling infection has been extensively studied in HIV and influenza patients^11–15^. The presence of ADCC Abs appears to be more critical than neutralizing Abs for controlling disease progression in HIV carriers^11,12^. Additionally, higher ADCC titers are associated with milder symptoms and lower viremia for influenza infection^14^. For DENV, ADCC activity has been demonstrated in patients’ serum samples^16^ and in pre-illness plasma samples^17^. Furthermore, the rise of NK cells in the peripheral blood of DENV patients at the early acute stage was shown to correlate with mild disease^18^, thus supporting a possible role of NK cells and ADCC in protection against severe diseases during natural DENV infection.

ADCC is initiated by the binding of Abs to infected cells, causing the cross-linking of the CD16 receptors and the triggering of degranulation and cytokine production of NK cells, which eventually leads to the elimination of the target cell itself. ADCC is a control mechanism for normal DENV infection, but we hypothesize that it is possibly a far more necessary control mechanism in the case of ADE. This is because when neutralizing Ab is not sufficient to fully neutralize the virus, heterologous secondary infection occurs. Since non-neutralizing Abs can cause ADE, therefore, possibly it is the infection in the ADE-affected cells which needs to be first eliminated by NK cell-mediated ADCC.

The main physiological target cells for ADE are peripheral blood monocytes^19^, macrophages and dendritic cells^7^. In this study, we first addressed if NK cells could be activated by ADE-affected monocytes, and secondarily, addressed the role of activated NK cells, including the role of IFN-γ and CD107a (surrogate ADCC activation) in counteracting ADE. We chose a culture system simulating secondary infection in peripheral blood by adding DENV and immune sera (autologous where possible) to whole peripheral blood mononuclear cells (PBMCs). Human PBMCs contain approximately 10% NK cells, with a majority of the cells expressing CD16, and also contain approximately 30% monocytes. Using the PBMC culture system we simultaneously monitored DENV infection, ADE, and activation of NK cells. Herein we demonstrate a possible protective role of ADCC Abs and NK cells activated under ADE conditions in suppressing ongoing and newly occurring ADE.

## Results

### Immune sera, but not naïve sera, resulted in ADE in monocytes either purified or unfractionated from whole PBMCs

**Figure 1:**
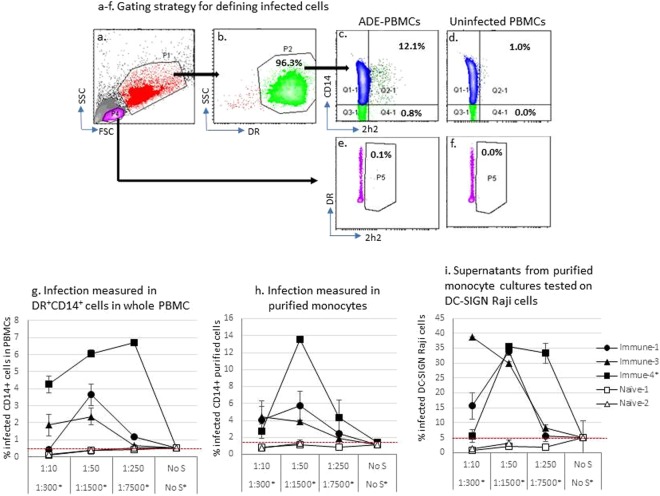
ADE in purified monocytes and whole PBMCs occurs in the presence of enhancement serum. PBMCs from one random blood donor were used as a source of monocytes and PBMCs for the ADE assays. ADE experiments were set up with whole PBMCs (**a**–**g**) or with purified CD14^+^ cells (**h**,**i**). Three immune and two naïve sera were diluted and added to donor cells together with DENV-1 virus. Serum dilution is shown on x-axis. “No S” is the virus-only control where cells were infected with virus only without serum. Panels a-f show the gating strategy for determining infection of DR^+^CD14^+^ monocytes in whole PBMCs. Gating and subgating are shown by the arrows. Infection was determined directly in monocytes (**g**,**h**) by co-staining surface markers DR (b)  and CD14 and then intracellularly with fluorescent 2H2 Ab (**c**–**d**). Viruses produced in the supernatants were tested using DC-SIGN Raji cells (**i**). Panel i shows the infection rate of Raji-DC-SIGN cells by supernatants collected from the same experiment of (**h**). The values presented in the figure g-i are absolute infection values where uninfected control was subtracted. All values are averages of duplicates. Error bars are standard deviation. The means of the peak ADE values in (**g**–**i**) were significant as compared to the virus-only control (marked as “No S”) tested by Student’s t test (p value were all <0.05). Data represents ≥3 experiments. The red dashed line indicates the infection with virus only without serum: values above the dashed line are considered to be ADE values.

ADE was performed with whole PBMCs (Fig. 1a–g). The characterization of the immune and naïve sera is shown in Table 1.

**Table 1:** Immune status of the serum samples.

Sample ID	Exposure to DENV	IgG ELISA to DENV1–4*	NT50**				ADE on K562 cells to DENV-1***
			DENV-1	DENV-2	DENV-3	DENV-4	
Immune-1	Yes	>1000	126	190	49	171	Positive
Immune-2	Yes	>1000	50	296	69	222	Positive
Immune-3	Yes	>100	<50	<50	268	<50	Positive
Immune-4	Yes	ND****	4351	43270	4358	9502	Positive
Naïve-1	No	<100	<50	ND	ND	ND	ND
Naïve-2	No	<100	<50	ND	ND	ND	Negative
Naïve-3	No	<100	<50	ND	ND	ND	Negative

*IgG titer measured by an in house IgG ELISA assay.

**Neutralizing Ab titer measured by a DC-SIGN Raji cell assay.

***ADE assessed by an in house K562 cell assay.

****ND = Not done.

The immune and naïve sera were serially diluted before being added to the monocytes together with DENV-1. Controls for ADE included monocytes infected with virus only without serum (denoted as “No S”) and uninfected cells. Figure 1a–f shows the gating strategy for interpreting the types of cells infected with DENV in the PBMCs. Two gates P1 and P4, based on forward scatter (FSC) and side scatter (SSC), depicted two distinctive populations, myeloid cells and lymphocytes/NK cells respectively in the unfractionated PBMCs (Fig. 1a). In P1, most of the cells were DR^+^ (P2, Fig. 1b). Within the total DR^+^ cells (P2), infection was mainly seen on cells expressing CD14 (Fig. 1c,d as the uninfected negative control). To be noted in the lymphocyte population gated in P4, little infection was seen regardless the status of DR expression (Fig. 1e,f as the uninfected negative control). For the DR^+^CD14^+^ subset, only the immune sera but not the naïve sera resulted in ADE (Fig. 1g). The background staining of 0.3% was already subtracted for Fig. 1g.

ADE was also performed with purified monocytes (Fig. 1h,i). In addition to measuring infectivity in monocytes, virus production in the culture supernatants was also quantified (Fig. 1i). The supernatants collected from the same experiment as Fig. 1h were tested on DC-SIGN Raji cells and the infection of Raji cells are shown in Fig. 1i. The background staining of 0.25% and 0.2% was subtracted for Fig. 1h,i respectively. Again, only the immune sera, but not the naïve sera resulted in infection enhancement (Fig. 1h,i).

### Kinetics of NK cell degranulation and IFN-γ expression

**Figure 2:**
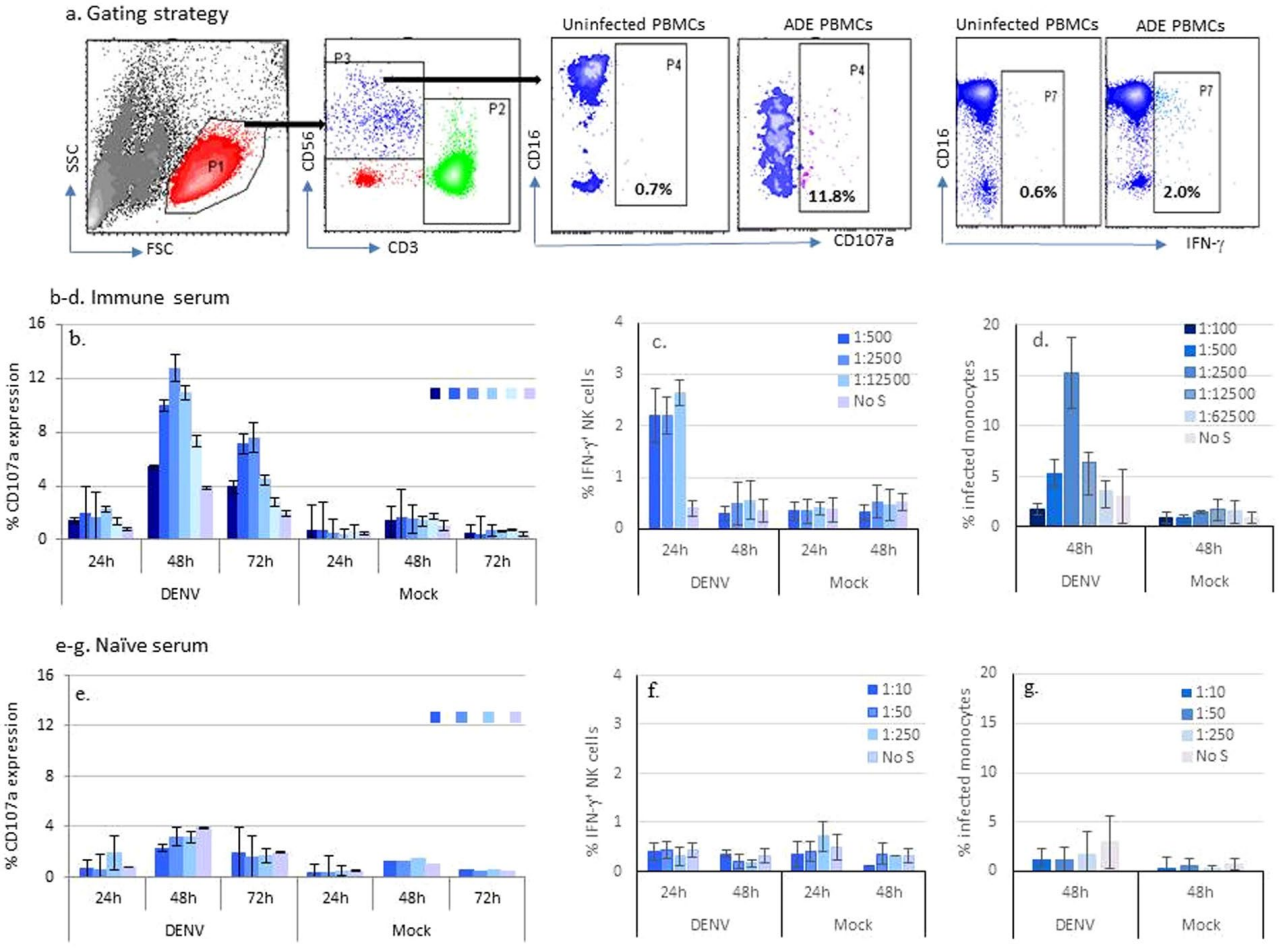
Kinetics of CD107a expression on NK cells from the PBMC-ADE cultures. Whole PBMCs from one random blood donor were used as source of PBMCs for the ADE/ADCC assay. (**a**) Gating strategy for CD3^−^CD56^+^ NK cells. One immune (Immune-4) serum (**b**–**d**) and one naïve (Naïve-1) serum (**e**–**g**) were diluted and added to donor cells together with DENV-1 virus or mock control. Levels of surface CD107a expression are shown (**b**,**e**). Levels of intracellular IFN-γ are shown (**c**,**f**). Infection was determined in CD14^+^ cells using fluorescent 2H2 Ab (**d**,**g**). Data is the mean of 3 experiments and the error bars are standard deviations of 3 experiments. No S = no serum.

Samples Immune-4 and Naïve-1 were then used to study the kinetics of NK cell activation in ADE/ADCC cultures. The sera and PBMCs were heterologous. For NK cells, the gate was drawn on CD56^+^CD3^−^ population (P3), and CD107a and IFN-γ expression were monitored on NK cells together with CD16 marker expression (Fig. 2a). NK cells showed relatively low expression of CD107a in cultures with DENV only without serum (denoted as “No S”) (Fig. 2b,e). The “No S” control indicated the level of direct activation of NK cells by infection alone but not by ADCC. CD107a expression increased in cultures treated with serially diluted immune serum (Fig. 2b). The mock controls were the cultures treated with no virus. NK cell degranulation peaked at 48 hours and remained elevated at 72 hours (Fig. 2b). CD107a expression on NK cells could be detected up to day 7 (data not shown). No elevated CD107a expression was seen with the naïve serum (Fig. 2e). IFN-γ expression in NK cells was measured intracellularly following the surface staining of the cocktail of CD3/CD56/CD16/CD107a (Fig. 2a) using the same Immune-4 and Naïve-1 sera. The kinetics of total IFN-γ expression is shown in Fig. 2c. IFN-γ production peaked at about 24 hours and declined to an undetectable level by 48 hours, and thus, IFN-γ staining was not repeated at 72 h (Fig. 2c). Again, IFN-γ response was only seen with the immune serum (Fig. 2c) but not with the naïve serum (Fig. 2f). The magnitudes of CD107a and IFN-γ expression corresponded to the magnitudes of infection (Fig. 2d). No ADE was seen with the naïve serum (Fig. 2g).

T cell activation was monitored by gating on CD3^+^ cells (P2) which showed a small subset of CD3^+^ CD56^+^cells (see Suppl Fig. 1). T cells were also activated to express CD107a with peak expression occurring at 48 hours post-infection, but the magnitudes were much lower as compared to NK cells (see Suppl Fig. 1). IFN-γ was not detected in T cells (data not shown).

CD107a and IFNγ expression were also monitored in relation to CD56 expression since a small proportion of NK cells are CD56^high^. The CD107a and IFNγ expression were mainly seen on CD56^low^ cells (data not shown).

### Degranulation and IFN-γ expression are mediated by different NK cells

**Figure 3:**
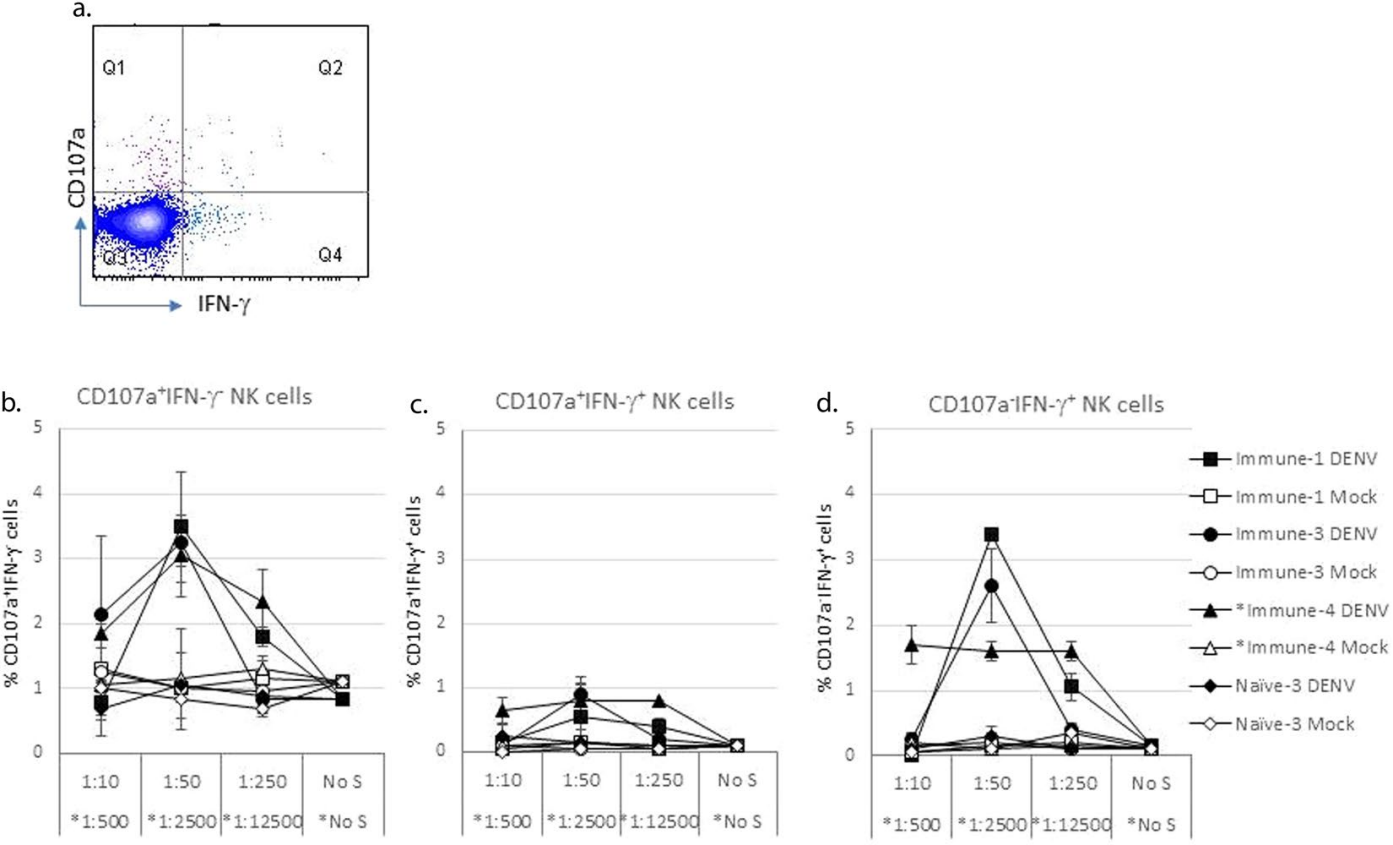
NK cells either express CD107a or IFN-γ under ADE/ADCC conditions. Three immune serum samples and one naïve serum sample were used for ADCC assay to quantitate NK cell expression of CD107a and IFN-γ. One random donor PBMC was used as the source of PBMCs. Cells were harvested after 24 hours of ADE culture and were first stained with surface markers and then fixed and permeabilized for intracellular staining of IFN-γ. Gating strategy for NK cells was the same as Fig. 2a. Functional NK cells were divided in quadrants as shown in panel a. Percentages of cells expressing only one marker CD107a (**b**) or IFN-γ (**d**) or co-expressing both markers (**c**) are shown. Data is one representative experiment chosen from three similar experiments. The mean and the standard deviation of the representative experiment are shown. No S = no serum.

We then asked if the IFN-γ and CD107a expressing cells were the same cells. Cell subsets were analyzed for expression of CD107a only, IFN-γ only, or both markers (Fig. 3a). IFN-γ and CD107a staining were performed for three immune (Immune-1, -3, -4) and one naïve (Naïve-3) sera (Fig. 3b–d) all at 24 hours. Base-line response was seen with the naïve serum (Fig. 3b–d). The majority of IFN-γ expressing NK cells were different from those expressing CD107a (Fig. 3b,d). A small percentage of NK cells co-expressed CD107a and IFN-γ (Fig. 3c).

### Cytokines produced by monocytes following DENV infection and co-culture with enhancement sera include IFN-γ

In order to assess the types of soluble factors produced by infected monocytes that may contribute to NK cell activation following ADE, ADE was set up with purified monocytes, and cytokines in the ADE supernatants were determined at 24 and 48 hours after infection. IFN-γ, IFN-α, TNF-α, TNF-β, IL-1Rα and IL-18 were detected following ADE infection (see Suppl Fig. 2). IL-12 and IL-15 were not detected (data not shown). Note that enhancement serum was removed after 3 hours of cell culture for monocyte ADE, therefore cytokines already present in the enhancement sera should not contribute to the baseline values.

### The requirements of optimal NK cell-activation, in the context of ADE, include infected target cells, soluble factors, and ADCC-inducing antibodies

**Figure 4:**
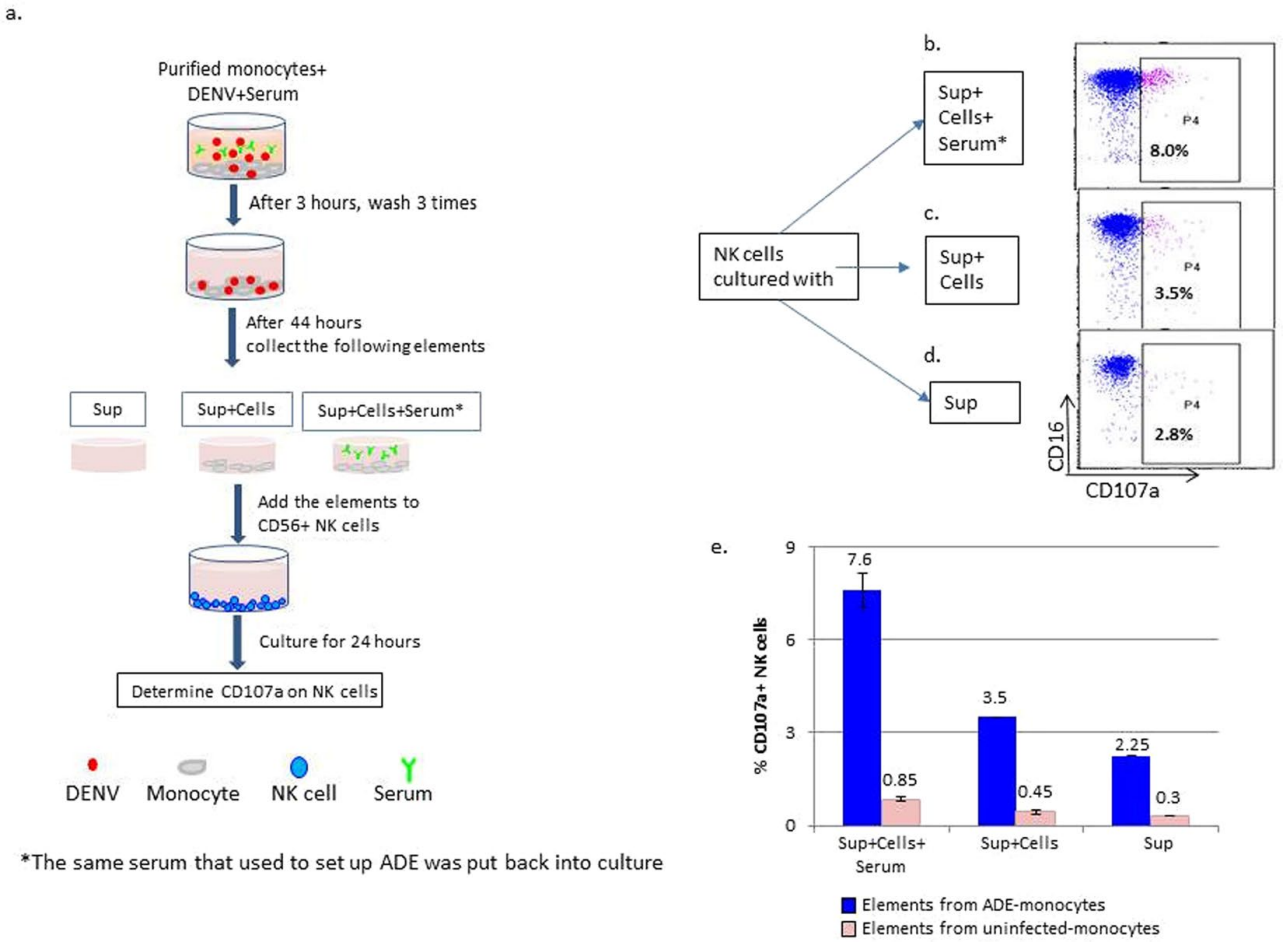
Requirement of supernatant, infected monocytes, and enhancement serum for optimal activation of NK cells. (**a**) Diagram showing the procedures of the experimental design. An ADE culture was set up using a random donor monocytes enriched with CD14-magnetic beads. The immune serum (Immune-3) at the dilution of 1:50 was added together with DENV-1 to the monocytes. After an initial 3 hours of incubation, the serum and virus unbound to the cells were washed out and the cultures were kept in fresh medium for 44 hours. An uninfected culture was set up as a negative control. From the monocyte ADE culture and the control culture, the following elements were collected: Sup (supernatant), Sup + Cells, and the same serum at the same dilution (Immune-3 at 1:50) was added back to some of the Sup + Cells (Sup + Cells + Serum). The collected elements were added to new cultures of freshly purified NK cells. After a 24-hour incubation, NK cell activation was determined by expression of CD107a. (**b**–**d**) Dotplots of NK cell activation after culturing with various elements collected previously. Showing in P4 is percentage of CD107a expressing cells. (**e**) Bar graph of the CD107a expression on NK cells cultured with various collected elements for 48 hours. Data is one representative experiment, done in duplicate cultures, chosen from three similar experiments. The mean and the standard deviation of the representative experiment are shown.

Since infected monocytes produce inflammatory cytokines, such as TNF-α and IFN-γ, which are known NK-cell activating cytokines, the elements in the monocyte ADE culture that could contribute to the activation of NK cells were investigated. To do so, monocytes were first enriched. As shown in the diagram Fig. 4a, enriched monocytes were cultured under the enhancement condition DENV + Serum (Immune-3 at 1:50). The unbound serum and virus were then washed out and the cultures were continued for about 44 hours in fresh culture medium. The supernatants (Sup) were harvested either alone (denoted as “Sup”) or in combination with cells (denoted as “Sup + Cells”). The same serum (Immune-3 at 1:50) used for setting up the original ADE culture was added back to some of wells with Sup + Cells, and were denoted as “Sup + Cells + Serum.” Elements from uninfected cultures were used as negative controls. The Sup, Sup + Cells, and Sup + Cells + Serum, were added to newly prepared NK cells enriched by negative selection using magnetic beads. The enrichment of NK cells was approximately 92%. As shown in Fig. 4e, both the Sup alone (Fig. 4d) and the Sup + Cells (Fig. 4c) activated NK cells to express CD107a compared to the negative controls. However, the highest activation was seen in NK cells cultured with all three elements Sup + Cells + Serum (Fig. 4b). NK cells cultured with elements collected from uninfected monocytes showed minimal activation with CD107a expression below 0.85% (Fig. 4e). The data indicate that both the soluble factors derived from infected monocytes and the physical contact between NK cells and infected monocytes can activate NK cells, but optimal activation requires the presence of ADCC Abs.

### ADE/ADCC with autologous serum and PBMCs and the function of NK cells for suppression of ADE

**Figure 5:**
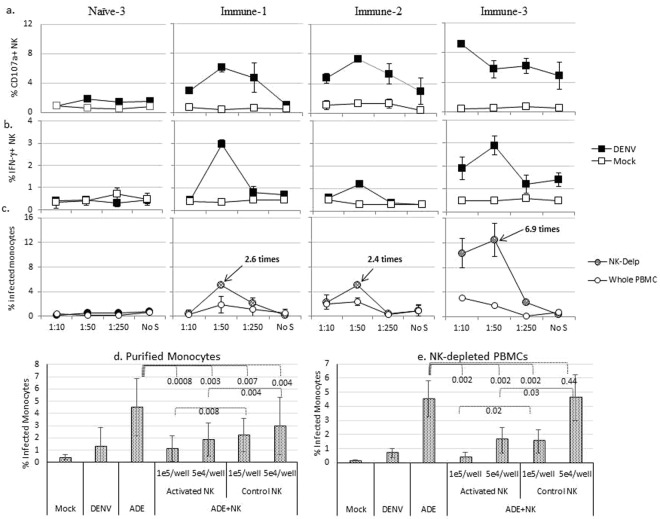
ADE and NK cell activation with autologous serum and PBMCs and ADE in the presence or absence of NK cells. Panels a-c were done with autologous serum and PBMCs. NK cell activation marker CD107a (panels a) was determined at 48 hours and IFN-γ (panels b) was determined at 24 hours post ADE. Panels c compares infection of CD14^+^ monocytes in ADE/ADCC experiments between whole PBMCs and NK-depleted PBMCs. For panels c, cells treated with serum only without virus had baseline 2H2 staining of less than 1% and the values were subtracted from the corresponding infection values. Therefore the data presented are absolute infection values. Data is one representative experiment of at least two experiments. Error bars are standard deviation of culture duplicates. Serum dilution is shown on the x-axis and “No S” = no serum. Panels d and e were done with a heterologous donor PBMCs. Panels d and e, PBMCs were treated with serum only (Immune-3) (for “Control NK”) or with serum + DENV-1 (for “Activated NK”) for 48 hours, and NK cells were purified. Purified NK cells were added back to new ADE experiments set up with purified monocytes (**d**) or with NK-depleted PBMCs (**e**) using the same enhancement serum (Immune-3 at 1:50). The x-axis shows the culturing conditions for the new ADE assays: Mock (uninfected), DENV (virus only), ADE (DENV + serum), and ADE + NK cells. The primed NK cells were added at the beginning of the new ADE experiments. After 48 hours, the % infected monocytes were determined. Data are means of 3 independent experiments. Error bars are standard deviation of 3 experiments. The difference between “ADE” and “ADE + NK cells” is shown by p values of Student’s t tests (see the numbers inbeded in **d** and **e**).

The kinetics study, described above, was done with sera and heterologous donor PBMCs. Monocyte ADE/ADCC was furthered monitored with autologous sera and PBMCs. CD107a (Fig. 5a) and IFN-γ (Fig. 5b) expression on NK cells was detected in autologous immune samples (Immune-1, -2, and -3), but not in the naïve sample (Naïve-2). ADE was detected in immune samples, but not in the naïve sample (Fig. 5c). NK cells expressed both degranulation and IFN-γ response in this autologous setting. The NK cell activity corresponded to ADE. The NK response and monocyte infection levels in Fig. 5 appeared to be different from Fig. 1 as different PBMCs (autologous *vs* donor PBMCs) were used.

Using the autologous PBMCs and sera, the antiviral function of NK cells was investigated. NK cells were depleted from whole PBMCs, and NK-depleted PBMCs were compared with whole PBMCs in terms of ADE. There was a marked increase of ADE in monocytes in NK-depleted PBMCs as compared to whole PBMCs (Fig. 5c).

The antiviral function of NK cells was also investigated in another assay in which the activated NK cells were isolated from an ADE/ADCC culture and added back to a new ADE experiment. Due to the limitation of the autologous PBMCs, this assay was done with heterologous serum and PBMCs. NK cells were isolated from the whole PBMCs treated with serum only or DENV + serum for 24 hours and were denoted as “Control NK” or “Activated NK” respectively). The NK cells were added to a new ADE experiment, assayed with either purified monocytes (Fig. 5d) or with PBMCs depleted of CD56^+^ cells (Fig. 5e). Infection of monocytes was examined after 48 hours of assay. The difference of ADE without and with NK cells was analyzed using Student’s t tests. For both ADE assays, infection was significantly suppressed by adding NK cells stimulated in a previous ADE experiment (activated NK). Infection was also significantly suppressed by “Control NK”. However, the suppression of ADE was significantly more profound by “Activated NK”. The inhibitory effect of activated NK cells in suppression of ADE was dose-dependent as more NK cells showed a greater suppression of ADE.

### Neutralizing IFN-γ expression decreases the anti-ADE function of NK cells

**Figure 6:**
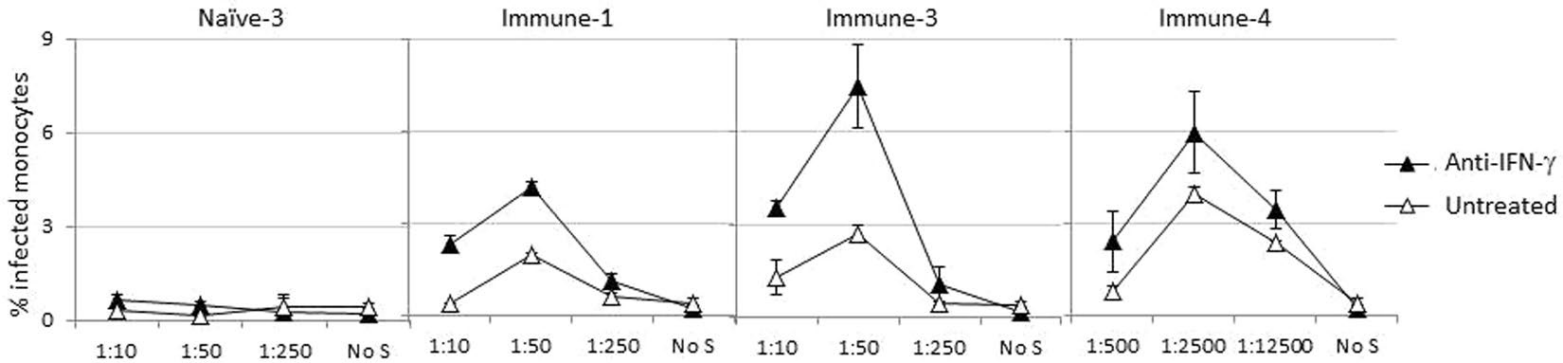
Anti-IFN-γ treatment increased ADE. PBMCs from one donor were used to set up ADE/ADCC assays with three immune sera and one naïve serum. PBMCs were cultured with DENV and serially diluted serum. Monoclonal Ab to IFN-γ was added at the beginning of the assay or cells were left untreated. After 48 hours, infection of CD14^+^ cells was determined by 2H2 staining. Data is one representative experiment chosen from three similar experiments. The mean and the standard deviation of the representative experiment are shown. No S = no serum.

Since NK cells produced IFN-γ, the role of IFN-γ in antiviral function of NK cells was investigated in the context of ADE using neutralizing monoclonal Ab against IFN-γ. Treating PBMCs with anti-IFN-γ increased the level of ADE in monocytes (Fig. 6), suggesting that part of the anti-ADE function of NK cells is achieved by IFN-γ−dependent mechanisms.

## Discussion

A large proportion of DENV infections are subclinical, suggesting that Abs in these subclinical cases did not block infection completely and that protection is not sterile. Therefore, besides Ab neutralization, other immune mechanisms which inhibit intracellular infection, such as ADCC, must have been employed to control and eliminate virally infected cells and therefore viruses. During a secondary infection, we hypothesized that the elimination of ADE-affected cells could be the major task early on for the host system via an ADCC mechanism.

Using PBMCs to study ADE *in vitro* could simulate ADE in the peripheral blood. The dynamic changes of the immune cells, especially NK cells, T cells and monocytes, that we observed may be a reflection of the changes in the peripheral blood during a secondary DENV infection. Primarily, ADE was considered to be a marker to predict clinical outcomes of secondary infection. Kliks *et al*. used PBMCs to quantitate ADE and showed that positive titers of ADE in pre-illness serum samples are associated with the development of symptomatic infections as opposed to asymptomatic infections in endemic children^5^. Laoprasopwattana *et al*. had a similar study design but used different material to quantitate ADE, a K562 cell line instead of PBMCs, and showed that ADE values did not correlate with disease severity^20^. The discrepancy of the previous ADE work suggested the importance of the intrinsic impact of ADE to the immune system. An increasing number of studies demonstrate that ADE can alter molecular signalling to weaken the antiviral response of the host cells. Understanding the intrinsic pathways of ADE ultimately may open unprecedented opportunities for future immune intervention to rescue the negative impact of ADE. Monocytes, macrophages and dendritic cells bear all three FcR on their cell surface and therefore are the most likely cell types for ADE *in vivo*^21^. In this study, we not only assessed ADE as a distinct phenomenon, but concurrently also focused on finding potent controls against ADE and the mechanisms to minimize the negative impact of ADE to the host system. Our study provides evidence that there are ADCC Abs in the same serum that causes ADE, and these ADCC Abs can activate NK cells to fight back against ADE.

We found a positive correlation between NK cell degranulation and IFN-γ production with the level of ADE in human PBMCs. This may be a perplexing observation as the antiviral function of NK cells should ensure less infection; therefore, higher NK cell degranulation and cytokine production should be associated with lower ADE. We suggest that ADE in the peripheral blood occurs before NK cells are optimally activated. Therefore, NK cells cannot eliminate the initiation of ADE, but once activated can control infection when infection continues. This is demonstrated in our NK-cell depletion experiment where ADE was set up with NK cell-depleted PBMCs. NK cell-depleted PBMCs had several times more infection compared to whole PBMCs, suggesting that NK cells did not completely eliminate but did reduce ADE. Further, we added activated NK cells back to a new ADE assay with enriched monocytes or with PBMCs depleted of NK cells, and the activated NK cells suppressed ADE, suggesting that activated NK cells can suppress newly occurring ADE. ADCC activity has been associated with protection against severe diseases for HIV and influenza infection^12,14^. Jegaskanda *et al*. reported that high ADCC Ab titer was associated with lower clinical symptoms and lower virus titers in a clinical challenge study with influenza virus H3N2 in subjects with no protective hemagglutination inhibition assay Ab titer^14^. Our *in vitro* data suggested that NK cells activated through ADCC can reduce the level of ADE through suppressing of ongoing or newly occurring infection.

We have demonstrated that the supernatant from the infected monocytes alone can activate the degranulation of NK cells. Clearly there is a role for both cytokines and direct cell-to-cell contact of NK cells and target cells for activation of NK cells. NK cells express IL-2R, IL-12R, IL-15R, IL-18R and IL-21R^22^. Previously, we reported that monocytes can produce type I IFN (IFN-α), TNF-α, and IL-10, but not IL-12, when infected with DENV through ADE^19^. In this study, we confirmed this previous observation and also saw production of TNF-β and IL-1Rα. A low level of IL-18 was detected. We did not detect IL-2, IL-12 or IL-15 in the supernatant of infected monocytes. Type I IFNs are an early and critical regulator of NK cell numbers, activation, and antitumor activity^23^. Others have shown that IFN-α can act on other cells, such as dendritic cells and monocytes, to produce IL-15 and IL-18, which promote NK cell proliferation during mouse cytomegalovirus^24^ or mucosal herpes simplex virus type 2 infections^25^. The cytokine profile suggested that the cytokines produced by ADE-infected monocytes responsible for activating NK cells possibly are type I IFN, TNF-α and IL-18.

IL-12 can be suppressed by Th2 cytokines and by FcR ligation^26^. Previous research showed that ligation of FcR on human monocytes suppresses IL-12 transcription and protein production in response to a variety of proinflammatory stimuli^26,27^. Others have found that ligation of macrophage FcR dramatically enhances IL-10 production in response to low amounts of lipopolysaccharides (LPS)^28^. The IL-10 produced by macrophages after FcR ligation is sufficient to completely inhibit the production of IL-12 p70^28^. This inhibition is not on the RNA level, and can be restored by exogenous IL-12^28^. Furthermore, type I IFNs inhibits dendritic cell IL-12 p40 expression^29^. Taken together, it is likely that the ligation of FcR during ADE and the cytokines IFN-α and IL-10 detected in ADE-affected monocytes suppressed IL-12 production. This could be an intrinsic mechanism that ADE modulates host immune response. Our future effort is to investigate if IL-12 production in ADE-affected monocytes can be restored, and if this IL-12 restoration can affect the production of IFN-γ by NK cells. Further experiments will be needed to define whether this regulation is also influenced through expression of NK cell activation/inhibition marker expression, as cytokines can regulate NK cell function through expression of these receptors/ligands.

We observed that the optimal activation for NK cell degranulation required infected monocytes, plus soluble factors in the supernatant, and plus serum, indicating that CD16 (and CD16-mediated recognition of Abs in sera) on NK cells plays an important role in NK cell activation. This further illustrates that inflammatory cytokines secreted by infected cells (mostly the myeloid cells) can contribute to the activation of NK cells, but the presence of ADCC Ab in the bulk of the enhancement serum is critical for this activation. Distinguishing Ab repertoire between ADE and ADCC is challenging but necessary. While ADE Abs might be predominantly specific to antigens on the surface of a viral particle, such as envelope and membrane (or pre-membrane) proteins, ADCC Abs may additionally target non-structural proteins. We have previously shown that infected DC-SIGN Raji cells express PrM and E antigens on the surface of infected cells^10^. We also detected surface expression of non-structural proteins using monoclonal Abs (data not shown). Moreover, secreted NS1 can bind back to the surface of uninfected cells primarily via interactions with heparan sulfate and chondroitin sulfate E on many types of epithelial and mesenchymal cells^30^. Abs to non-structural protein antigens on the surface of infected cells are possibly ADCC Abs with no enhancement activity, and these Abs may be of great interest for studying ADCC. Further characterization of DENV antigens expressed on the surface of infected cells may reveal more information on the Ab repertoire for ADCC.

As discussed above, NK cells activated in our culture system inhibited viral infection. When IFN-γ was neutralized, the suppression of ADE was weakened. We showed that NK cells could secret IFN-γ upon activation (Figs 2 and 3). We also observed a decrease in the total amount of IFN-γ in the culture supernatants of NK-depleted PBMCs as compared to whole PBMCs under the ADE condition (data not shown). IFN-γ production is another important function of NK cells besides degranulation. IFN-γ is a potent antiviral cytokine. Hence, our data suggests that the inhibition of viral infection is partly achieved by IFN-γ secreted by NK cells. The kinetics of degranulation and IFN-γ response were different. NK cell degranulation peaked at 48 hours and was still detectable more than one week after infection; whereas IFN-γ was short lived, detectable at 24 hours but not detectable at 48 hours post-infection. Additionally, we provide evidence that the majority of NK cells either responded with IFN-γ secretion or degranulation, not both. IL-12/STAT4 is critical for NK cell IFN-γ expression, whereas IFN-αβ/STAT1 is required for induction of cytotoxicity. Furthermore, it should be noted that the proliferation of NK cells is STAT4-independent but dependent on IFN-αβ/STAT1 induction of IL-15^22,31^.

In conclusion, enhancement of DENV infection in human monocytes occurs in the context of activating NK cells. The activation of NK cells was partially mediated by cytokines, possibly the type I IFN and IL-18 produced by infected monocytes. IL-12 and IL-15 were not produced by ADE-affected monocytes. The optimal activation of NK cells relied on the presence of ADCC Abs. The NK cell functions, degranulation and IFN-γ production, showed different kinetics: degranulation peaked at 48 hours and persisted for 7 days, whereas IFN-γ peaked at 24 hours and dropped to undetectable level within 48 hours. Activated NK cells suppressed ongoing and newly occurring ADE and the mechanism involved IFN-γ. The detailed regulatory mechanism of NK cell activation, in the context of cytokines and activation/inhibitory markers, requires further investigation. This study emphasizes the critical role of ADCC Abs in the polyclonal non-neutralizing/poorly neutralizing serum for counteracting ADE and protection from disease severity during a secondary DENV infection.

## Materials and Methods

### Sera and PBMC samples

The study was conducted using protocols approved by the Naval Medical Research Center Institutional Review Board and the Walter Reed Army Institute of Research Institutional Review Board in compliance with all applicable Federal regulations governing the protection of human subjects. De-identified human sera and PBMC samples were used. Informed consents were obtained from all study participants. All samples were tested by ELISA for IgG titers and neutralizing (Neut) Ab titers against DENV-1 using methods published previously^32^. Immune and naïve status of a sample was determined based on the sample’s ELISA endpoint titer (the lowest serum dilution giving a positive reading) or Neut titer. PBMCs were isolated using gradient centrifugation on Ficoll-paque (GE Healthcare Life Sciences, Pittsburgh, PA) using the manufacturer’s protocol. PBMCs were kept frozen until use. Based on the ELISA IgG and Neut titers, 4 immune and 3 naïve pairs of serum/PBMCs were chosen. The four immune samples were numbered as Immune-1, -2, -3, and -4. The three naïve samples were numbered as Naive-1, -2, and -3. The characteristics of the samples: exposure history (self-reported), anti-DENV IgG, neutralizing and ADE Ab, are shown in Table 1. ELISA was performed using an in house standard protocol. The coating antigen for ELISA was a mixture of DENV-1, -2, -3, and -4 purified virus. The neutralization Ab was measured to all 4 serotypes using DC-SIGN Raji cells as described previously^19^. ADE to DENV-1 was measured on K562 cells as previously described^33^. All 4 immune sera had self-reported previous exposure, ELISA IgG titer >100, higher heterologous neutralizing Abs titers than that of to DENV-1, and positive ADE activity. All 3 negative samples had ELISA IgG titer <100, neutralizing Ab titers <50, and negative ADE activity.

### Virus strain

DENV-1 West pac 74 was used for the study^34^. The virus was propagated in Vero cells and titrated by Vero cell plaque assay following standard operating procedures from our lab^34^. The titer of the stock virus was 6.8 × 10^6^ plaque forming units (PFU)/mL. The virus stock was thawed and 1:2 diluted before use. A standard dose of 70 µl/2 × 10^5^ cells/well of working dilution virus was used for all experiments. The multiplicity of infection (MOI) is 1.2.

### Culture medium

The culture medium (CM) was RPMI 1640 w/o L-Glutamine supplemented with 1% Penicillin 10,000 IU/mL and Streptomycin 10,000 µg/mL (Invitrogen, Carlsbad, CA), 1% L-glutamine 200 mM in 0.85% NaCl, 1% non-essential amino acid (all from Mediatech, Manassas, VA) and 10% heat-inactivated foetal bovine serum (FBS) (Quality Biologicals, Gaithersburg, MD).

### ADE assay with purified monocytes

We have previously published an ADE assay using purified monocytes^19^. Briefly, PBMCs were thawed and monocytes were enriched using CD14-magnetic beads (Miltenyi Biotec, Bergisch Gladbach, Germany). The enriched monocytes were placed in a U-bottom 96-well plate (Corning Incorporated, Corning, NY) at 2 × 10^5^ cells/well in CM in a 37 °C humidified CO_2_ incubator overnight to allow the recovery of frozen cells. The next day, the monocytes were centrifuged once and the media was replaced with fresh CM, and the cells were ready for setting up the ADE assay. To set up an ADE assay, serially diluted immune and naïve sera were added to the monocytes together with the virus. The ADE wells contained 30 µL of serum and 70 µL of virus per well. Control wells included serum-treated but uninfected, serum-untreated but infected, and serum-untreated and uninfected. After 3 hours of incubation in the humidified 37 °C 5% CO_2_ incubator, the cultures were washed with plain RPMI 1640 3 times to remove unbound virus and serum. After the last wash, the cells were placed in 150 µL/ well CM and were cultured for about 44 hours. Then the monocytes were washed, fixed and permeabilized using Perm-Fix and Perm-Wash buffers (BD Biosciences, San Jose, CA) and stained with a fluorescent monoclonal antibody 2H2 specific to DENV Pre Membrane (PrM). The percentages of infected monocytes were determined on a FACS CANTO II (BD Biosciences). Infection percentage was determined by comparing the percentages of 2H2-positive cells between the infected cultures and the corresponding uninfected cultures (background control). ADE was determined by comparing the infection percentages between cultures treated with enhancing serum and cultures not treated with enhancing serum. ADE was also assessed in the monocyte culture supernatants. Culture supernatants 50 µl/well were collected and added to DC-SIGN Raji cells (1.2 × 10^6^ cells/60 µl/well) as previously described^19^. The supernatants were cultured with DC-SIGN Raji cells overnight and the infection percentages of DC-SIGN Raji cells were determined.

### ADE/ADCC assay using whole PBMCs

ADE/ADCC assay was performed by adding serum and virus directly to whole PBMCs and then by monitoring monocytes and NK cells for infection and cell activation simultaneously. Based on our previous observation, we hypothesized that ADE and ADCC Abs coexisted in the same serum. It should be noted that during the whole ADE/ADCC assay period, the serum was not washed out but maintained in the cultures for the whole assay period. This would allow the ADE Abs to first enhance infection of FcR-bearing cells, and then the ADCC Abs to recognize infected cells to trigger ADCC.

Briefly, whole PBMCs were thawed and placed in a U-bottom 96 well tissue culture plates (Corning Incorporated, Corning, NY) at 2 × 10^5^ cells per well in 50 µL of CM and kept overnight in the 37 °C 5% CO_2_ humidified incubator to allow cells to recover from thawing. The next day, before setting up the ADE/ADCC assay, the cells were centrifuged once and the CM was replaced with fresh CM. Serially diluted immune or naïve sera were added to the plate, as well as the virus. The ADE/ADCC cultures contained 30 µL/well of serum and 70 µL/well of virus. The sera and PBMCs were autologous or heterologous depending on the experiment design. The cultures were kept for up to 7 days. Infection was determined in CD14^+^ cells using the fluorescent monoclonal Ab 2H2. Briefly, PBMCs were stained for surface expression of DR, CD16 and CD14 and then fixed, permeabilized, and stained intracellularly with 2H2. Infection was monitored at 24, 48, and 72 hours.

Expression of CD107a and IFN-γ from NK cells were monitored in the same set of cultures. Surface expression of CD107a on CD3^−^CD56^+^ NK cells was determined using an Ab cocktail CD16/CD3/CD56 /CD107a (all from BD Biosciences) daily for up to 3 days and on day 7. The CD107a expression was done on un-fixed cells. Briefly, the PBMC plate was washed with PBS once and stained with the cocktail for 30 minutes. The plate was washed twice with PBS and percentages of CD107a^+^ NK cells (CD3^−^CD56^+^) was determined on a FACS CANTO II flow cytometer. The expression of IFN-γ was determined intracellularly using the above cocktail as surface markers and anti-IFN-γ (clone 4 S.B3, BD Biosciences) as an intracellular marker. Prior to staining, Golgi Plug (BD Biosciences) was added at the final dilution of 1:1000 to the cultures for 4 hours. The plate was washed and stained with surface Abs and was then fixed, permeabilized, and stained with anti-IFN-γ Ab.

### Analyzing supernatant, cells, and serum in the ADE culture for activation of NK cells

In order to find the factors in the ADE culture necessary for NK activation, we purified monocytes to set up ADE cultures. We then collected the supernatants and monocyte cells separately and added these elements to unstimulated NK cells with or without the same serum that caused ADE. We then investigated which of these elements influenced NK cell degranulation.

Briefly, a serum at an indicated dilution was added to purified monocytes together with the virus. After the first 3 hours of incubation, the unbound virus and serum were washed out and the cultures were placed in fresh CM. After an additional 40–44 hours of incubation, supernatants and cells were harvested separately and were added to NK cells. The monocytes and the NK cells were from the same donor. The NK cells were freshly prepared using a Dynabeads Untouched Human NK Cell Kit (Thermo Fisher Scientific, Waltham, MA) according to the manufacture’s protocol. The supernatants (denoted as “Sup”), a combination of monocyte cells and supernatants (denoted as “Sup + Cells”) and another combination of monocyte cells and supernatant and serum (denoted as “Sup + Cells + Serum”) were added directly to the enriched NK cells. Expression of CD107a was examined on NK cells after 24 hours of the co-culture. The experiment design is also illustrated in Fig. 4a. Controls for this assay included supernatants and cells from uninfected monocytes, infected monocytes without enhancement serum, and uninfected but serum treated monocytes.

### Cytokines in monocyte ADE cultures

A Th1/Th2 cytokine 18-plex human ProcartaPlex kit was used to assess the cytokines in the supernatants of the ADE-affected monocytes. The 18-plex cytokines include IL-1β, IL-2, IL-4, IL-5, IL-6, IL-7, IL-12p70, IL-13, IL-31, IL-1Rα, IFN-γ, GM-CSF, TNF-α, IFN-α, TNF-β, IL-1α, IL-15 and IL-18 (Thermo Fisher Scientific). The assay was performed following the manufacturer’s protocol.

### Depletion of NK cells from the PBMC ADE cultures and addition of NK cells back to ADE cultures

To investigate the function of NK cells in suppressing ADE, NK cells were depleted from whole PBMCs using the CD56^+^ magnetic bead kit (Miltenyi Biotec) according to the manufacturer’s protocol and the depletion achieved to >95%. ADE was set up with intact PBMCs and the NK-cell-depleted PBMCs and results were compared.

To further demonstrate the function of NK cells in suppressing ADE, we added the activated NK cells back to a new ADE assay set up with purified monocytes or PBMCs depleted of NK cells. Briefly, whole PBMCs were treated with serum only (for Control NK) or serum + virus (for Activated NK) as described previously for 24 hours. NK cells were then purified from the whole PBMC cultures using a Dynabeads Untouched Human NK Cell Kit (Thermo Fisher Scientific) and were added back to the new ADE assay. The new ADE assay was set up with either purified CD14 cells or with NK-depleted PBMCs. The cells were either uninfected (Mock), or infected with virus only (DENV), or infected with virus in the presence of ADE serum (ADE). The serum was not washed out. The NK cells were added at the beginning of the ADE cultures. Infection in monocytes was determined after 48 hours of ADE assay using the 2H2 monoclonal Ab. Infection was compared between cultures supplied with NK cells and cultures without NK cells.

### Neutralizing IFN-γ in the ADE/ADCC cultures

IFN-γ was neutralized in the whole PBMC ADE/ADCC assays. Briefly, monoclonal Ab to human IFN-γ (clone 1-D1K, Mabtech, Stockholm, Sweden) was dialyzed overnight in PBS and sterilized by filtering through a 0.22 μm PVDF syringe filter. The monoclonal Ab was added to the ADE/ADCC cultures at 10 µg/ml final concentration to neutralize the IFN-γ response. ADE was then compared between cultures with and without anti-IFN-γ Ab.

### Data analysis

Infection was presented using percentages of cells positively stained with 2H2 Ab (% 2H2^+^ cells). The uninfected cultures treated or not treated with sera served as background controls. ADE was determined using the percentages of infection in cultures containing enhancement serum above the percentage of infection without serum.

NK cell activation was presented using percentages of NK cells expressing CD107a and/or IFN-γ within the CD56^+^CD3^−^ cell population. Since NK cells can be activated in response to infection directly, the ADCC activity was determined as the % of CD107a^+^ NK cells in the presence of serially diluted serum above the “No serum” control. Statistics were performed using GraphPad Prism software (GraphPad; La Jolla, CA). Student’s T-test was used to analyze group differences.

## Supplementary information

Dataset1

## Data Availability

The datasets generated during and/or analyzed during the current study are available from the corresponding author on reasonable request.
