# Supplementary material for: NK Cells Activated through Antibody-Dependent Cell Cytotoxicity and Armed with Degranulation/IFN-γ Production Suppress Antibody-dependent Enhancement of Dengue Viral Infection
Source: Sci Rep. 2019 Feb 1;9:1109. doi: 10.1038/s41598-018-36972-2 (PMC6358599; doi:10.1038/s41598-018-36972-2)
Supplement: Supplementary file 1 — Dataset1 [file 41598_2018_36972_MOESM1_ESM.pdf]

NK Cells Activated through Antibody-Dependent Cell Cytotoxicity and Armed with Degranulation /IFN-γ Production Suppress Antibody-dependent Enhancement of Dengue Viral Infection

Peifang Sun[1]*, Maya Williams[2], Nishith Nagabhushana[1], Vihasi Jani[1], Gabriel Defang[2], Brian J. Morrison[2]

[1]Henry Jackson Foundation, Bethesda, MD, USA

[2] Viral and Rickettsial Diseases Department, Infectious Diseases Directorate, Naval Medical Research Center, Silver Spring, MD, USA

*Corresponding author: peifang.sun2.ctr@mail.mil

Supplementary Figure 1

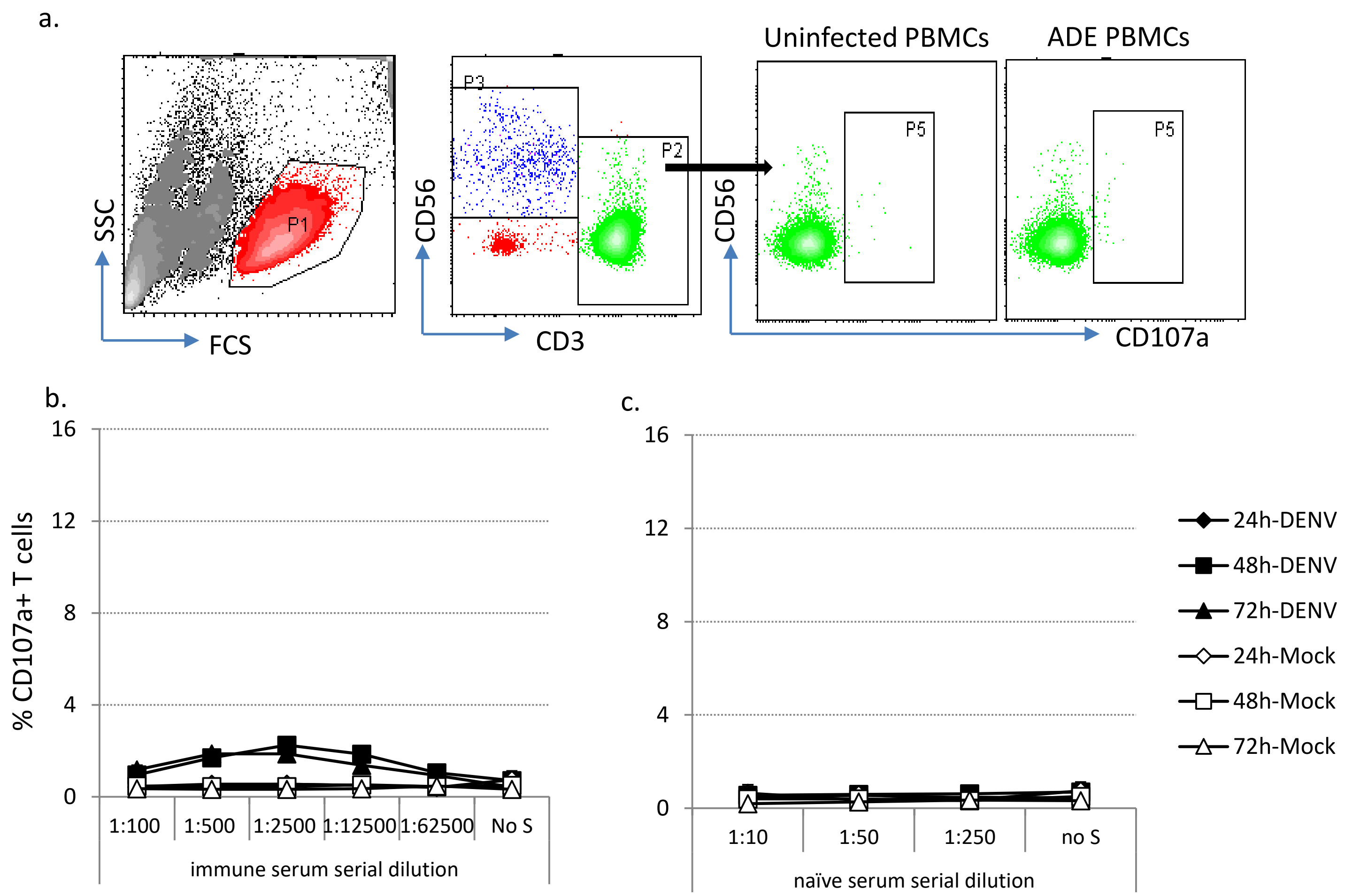

Supplementary Figure 1. Kinetics of CD107a expression on T cells from the PBMC-ADE cultures. Whole PBMCs from one donor were used as source of PBMCs for the ADE/ADCC assay. Sera from Immune-4 (b) and Naïve-1 (c) were diluted and added to donor cells together with DENV-1 virus. CD107a was determined on T cells using the gating strategy shown in panel a. Levels of CD107a expression are shown (b, c). Data is the mean of 3 experiments and the error bars are standard deviations to 3 experiments. No S = no serum.

Supplement Figure 2

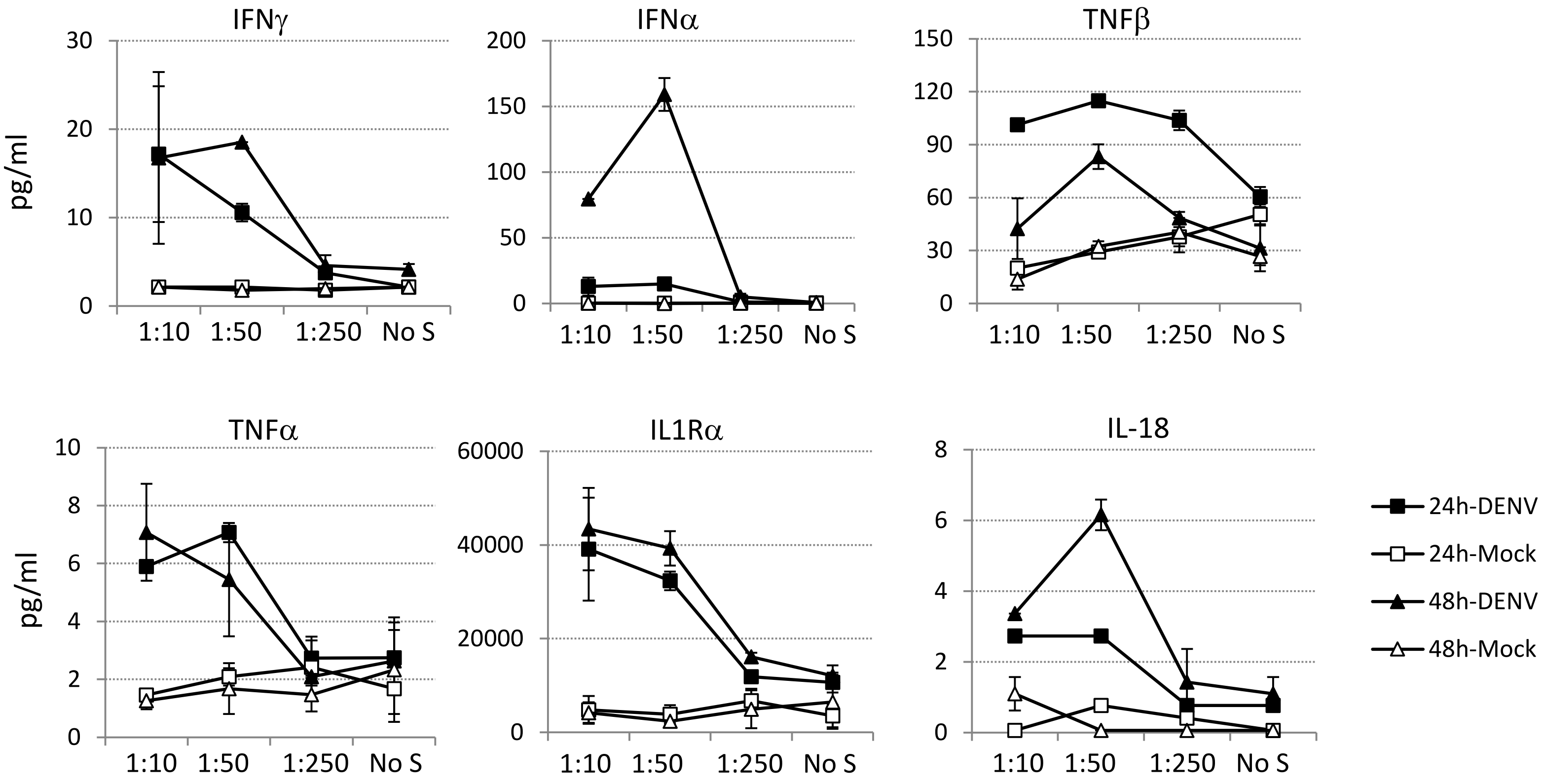

Supplementary Figure 2. Increased cytokines detected in monocyte cultures following DENV infection with enhancement sera. PBMCs from a random donor were used as the source for monocytes. Data from one immune serum is presented. Purified monocytes were cultured with serially diluted serum and DENV. After 3 initial hours, the cultures were washed three times to remove residue virus and serum, and fresh culture medium was added to maintain the cell culture. Cytokines in the culture supernatants were determined at 24 hours and 48 hours. Data is from one experiment and the error bars are standard deviation of culture duplicates. Results are representative of 2 experiments and 3 immune sera. No S = no serum.
